# Longitudinal activity monitoring and lifespan: quantifying the interface

**DOI:** 10.18632/aging.206106

**Published:** 2024-09-09

**Authors:** Su I Iao, Poorbita Kundu, Han Chen, James R. Carey, Hans-Georg Müller

**Affiliations:** 1 Department of Statistics, University of California, Davis, CA 95616, USA; 2 Department of Entomology, University of California, Davis, CA 95616, USA

**Keywords:** age-at-death, force of mortality, functional data analysis, longitudinal data, mediterranean fruit fly

## Abstract

Understanding the relationship between activity over the entire lifespan and longevity is an important facet of aging research. We present a comprehensive framework for the statistical analysis of longitudinal activity and behavioral monitoring and their relationship with age-at-death at the individual level, highlighting the importance of advanced methodological approaches in aging research. The focus is on animal models, where continuous monitoring activity in terms of movement, reproduction and behaviors over the entire lifespan is feasible at the individual level. We specifically demonstrate the methodology with data on activity monitoring for Mediterranean fruit flies. Advanced statistical methodologies to explore the interface between activity and age-at-death include functional principal component analysis, concurrent regression, Fréchet regression and point processes. While the focus of this perspective is on relating age-at-death with data on movement, reproduction, behavior and nutrition of Mediterranean fruit flies, the methodology equally pertains to data from other species, including human data.

## INTRODUCTION

In recent years there has been a surge in the availability of complex longitudinal data capturing various aspects of organismal life history. Relating such data to individual lifespan and age-at-death is of paramount interest for the study of aging and longevity. We provide an overview of advanced statistical methodologies that are particularly well-suited for analyzing such data, with a focus on understanding the complex relationships between age-at-death and activity, reproduction and diet at the individual level. While we illustrate the methodology with data for lifetime monitoring of Mediterranean fruit flies, it is generic and easily adapts to other species. The methods are interpretable and enjoy broad applicability, including for human longitudinal aging and activity monitoring data [1–5].

The longitudinal monitoring data we consider here encompass three key areas of life history: Activity (quantified as counts of movements per time unit) [6], reproduction (quantified as egg-laying per day for female flies) [7] and longitudinal behaviors (quantified as frequency of each behavior per time unit) [8]. Of central interest is the prediction of remaining lifespan for an individual who has survived to a current age *a*, based on the available life history (activity, diet etc.) data from birth to *a* for the individual, i.e., the longitudinal monitoring data available in the time interval [0, *a*]. Predicting the exact remaining lifespan for an individual at current age *a* is futile, but it is possible to predict the distribution of remaining lifetime for an individual [9] by utilizing Fréchet regression [10], as we demonstrate below.

Of interest is also the modeling of the longitudinally observed activity or egg-laying data to elucidate patterns and modes of variation of activity, using covariance and functional principal component modeling [11–13], where we specifically demonstrate the applicability of product functional principal component analysis [14] to jointly model two components of the activity data, intra-day activity measured in hours within each day and age-dependent activity, where age is measured in days, giving rise to a function-valued stochastic process [6].

Providing another perspective, we demonstrate the modeling of daily egg-laying data for female medflies with a global Cox point process model [15] that can be coupled with a functional linear model [16] to quantify the relationship between longevity and reproductive activity [7, 17]. To study the effect of the current level of reproduction on the immediate reproductive rate, we demonstrate below the application of a concurrent regression [18] approach.

Another type of important life history data in aging research are age-varying behavioral data, where several categories of behaviors are observed simultaneously and one aims to establish patterns of behavior change as individuals age [8, 19–21]. Riemannian functional principal component analysis [22] is shown to be uniquely suited or the simultaneous longitudinal study of several behavioral components, accounting for the complex compositional and longitudinal nature of such data.

We note that these approaches can all be adapted to human longitudinal aging data when available, offering a statistical toolkit for researchers investigating human aging and longevity. The statistical methods for the most part are grounded in functional data analysis, which aims at flexible modeling of trajectories and longitudinal data [23–26], allowing for biological interpretations and facilitating the detection of patterns of aging and their relation with longevity.

## RESULTS

### Activity monitoring, diet and longevity

#### Movement activity profiles of mediterranean fruit flies

We illustrate the analysis of activity patterns and their relationship with diet and longevity with the movement activity profiles of 96 female adult Mediterranean fruit flies (medflies). The experiment utilized the Monitor-LAM25 system to obtain repeated observations of the 24-hour locomotory activity for the 96 medflies, where each fly was fed with one of three agar-based gel diets that differed in their sugar and yeast hydrolysate content (50%, 20% and 10% and labeled C50, C20 and C10). Each diet group consisted of 32 female medflies. Each fly was placed in its own glass tube (25 mm diameter, 125 mm length) and the activity count, defined as the number of times a fly passed through an infrared beam placed in the middle of the tube, was recorded for each minute until the death of the fly; a full description with further details can be found in Chen et al. [6].

#### Predicting remaining lifetime

Predicting the exact remaining lifetime for an individual still alive at a specific age *a* is futile due to the high random variation of individual age-at-death. A more feasible target is to predict the distribution of remaining lifetime for an individual alive at age *a*. We demonstrate this by demonstrating the effect of diet by predicting the distribution of remaining lifetime with Fréchet regression (see Appendix) for individuals in each of the diet groups C50, C20 and C10, where the predicted distribution of remaining lifetime is conditional on an individual still being alive at age *a*, where *a* varies. Of course, the larger the value of *a*, the more compressed the remaining lifetime distribution is, as remaining lifetime is becoming shorter with increasing age *a*.

**Figure 1:**
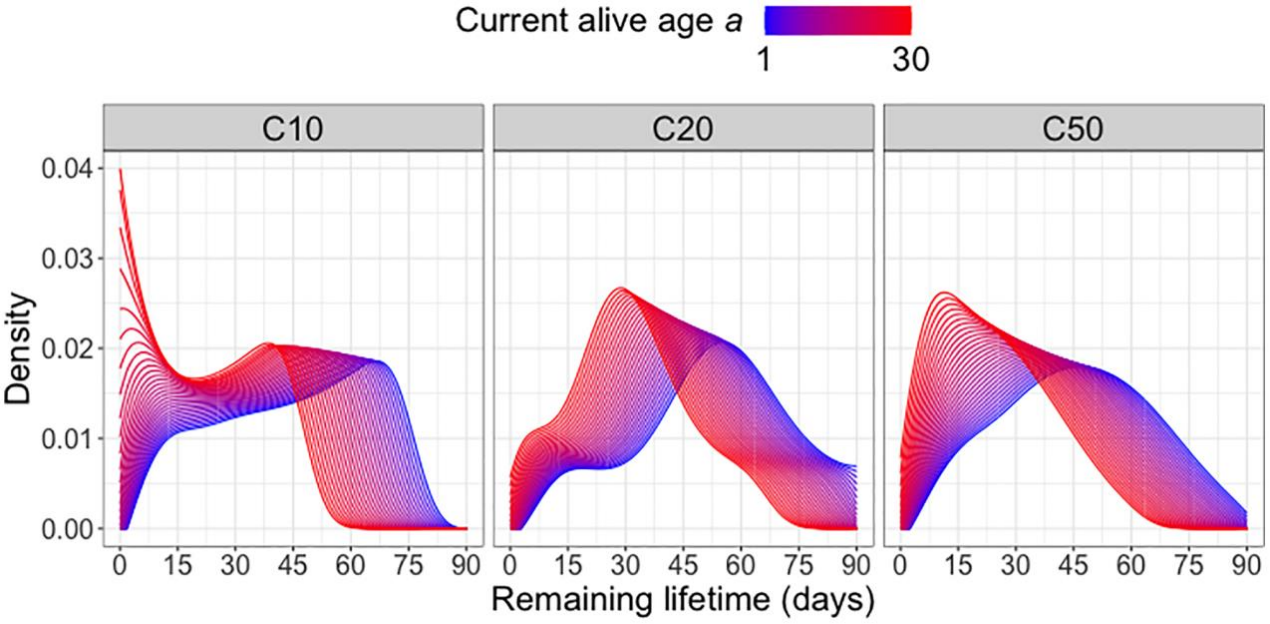
**Remaining lifetime distribution for different diet groups.** Predicted remaining lifetime distributions (visualized as densities) at different values of current alive age *a*, where a varies from *a* = 1 (blue) to *a* = 30 (red), for three different diet groups C10, C20, C50 (yeast hydrolysate content 10%, 20% and 50%), using global Fréchet regression (4) (see Appendix).

The target to be predicted is the remaining lifetime distribution at current age alive *a*, while the predictors are (*a, C*20*, C*50), in addition to current age alive *a* these are indicators for diet groups *C*20 and *C*50, where *C*20 = 1 if a fly is in diet group C20 and otherwise *C*20 = 0 and analogously for *C*50; if both *C*20 = 0 and *C*50 = 0, the fly is in diet group *C*10. The results for the predicted remaining lifetime distributions are shown in terms of the densities of these distributions in Figure 1. As expected, the densities of the predicted remaining lifetime distributions shift to the left as current age alive *a* increases, consistent with the expectation of shorter remaining lifetimes for older individuals. This pattern is observed for all diet groups, however there are variations in the magnitude and shape of the shift. Specifically, in the C10 diet group, the density peak shifts from approximately 70 days at *a* = 1 (i.e., right after eclosion) to around 40 days as the current age alive *a* increases to 30 days. At *a* = 30 days, the density values for the next 10 days shoot up rapidly, indicating elevated risk of death within the first 10 days after surviving past 30 days.

**Figure 2:**
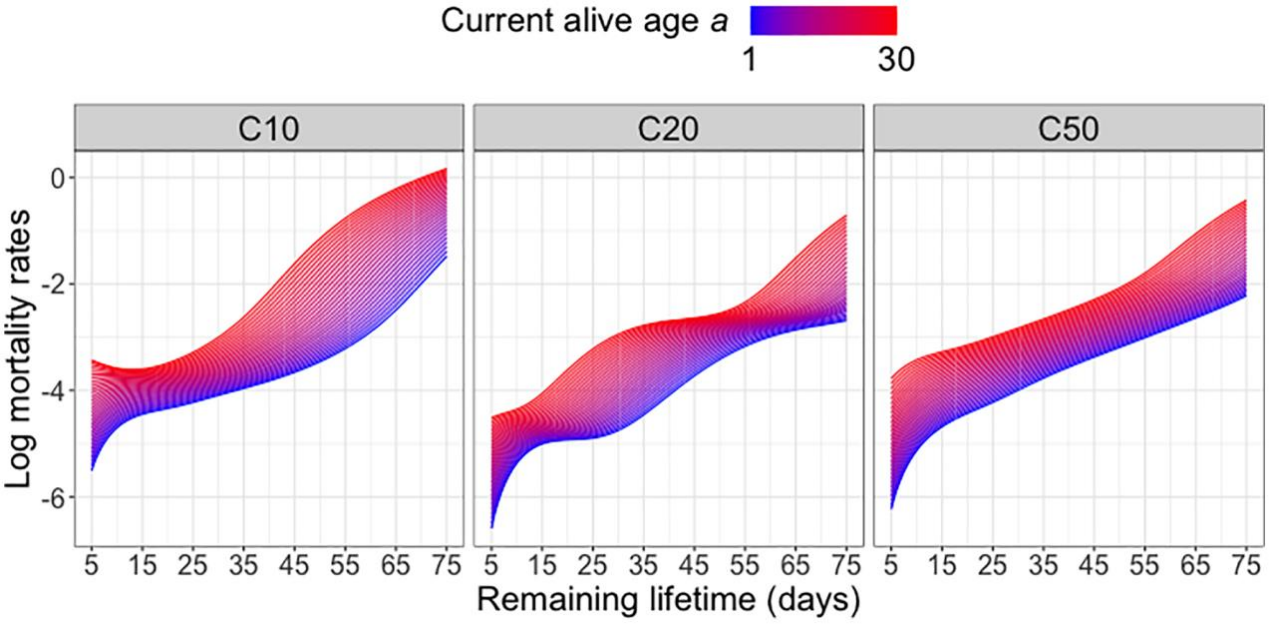
**Log mortality rates for different diet groups.** Log mortality rates (log hazard functions) of predicted remaining lifetime distributions across varying current alive ages *a* at which a subject is still alive for different diet groups; see equation (4) in the Appendix.

Predicted remaining lifetime distributions can be equivalently visualized as mortality rate (force of mortality, hazard function). Hazard functions can be derived from the density functions of the remaining lifetime distributions or alternatively directly estimated from lifetable data, with proper adjustments for right tail estimation [27, 28]. Figure 2 illustrates that, as expected, the mortality risk (represented by higher values of the log hazard function) increases with increasing current age alive *a* for all diet groups. Across all diet groups, higher ages alive *a* are associated with elevated mortality risk at all subsequent ages, in line with expected increased mortality risk in older populations. Among the three diet groups, the C10-fed medflies are subject to the highest mortality risk at older ages *a*, while the C20-fed medflies are seen to have a relatively lower mortality risk. However, the overall patterns of mortality risk across diet groups do not show substantial differences.

**Figure 3:**
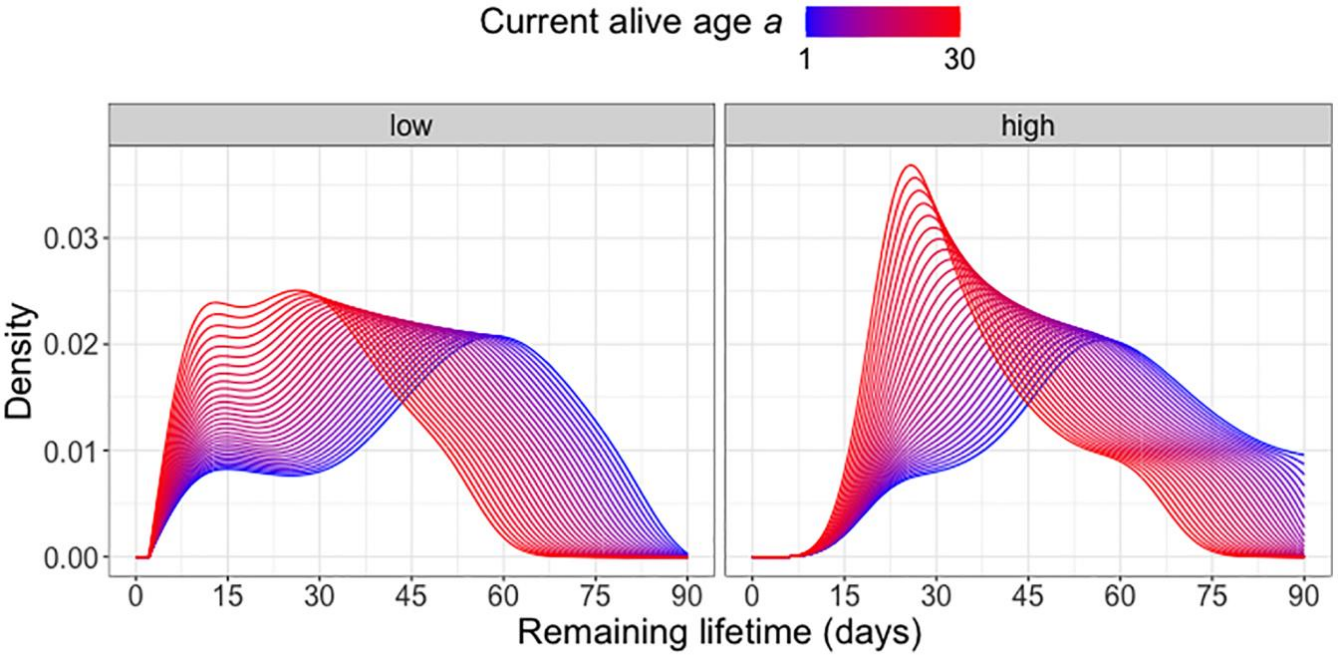
**Remaining lifetime distribution for different activity levels.** Predicted remaining lifetime distributions (visualized as densities) at varying alive ages *a* at which a subject is still alive and low (below median)/high (above median) activity levels, using global Fréchet regression (see (4) in the Appendix). Only C20-fed (yeast hydrolysate content 20%) flies are included in the analysis.

Focusing on the diet group C20, which according to Figure 2 is associated with the lowest mortality risk, we further examine the effect of current alive age *a* on the remaining lifetime distribution when also adjusting for the overall movement activity from time 0 until age *a*. Within each cohort surviving beyond age *a*, we classify the subjects into “high” (above median) or “low” (below median) activity groups and implement global Fréchet regression (see (2) in the Appendix) with the remaining lifetime distribution as response and predictors *a* and activity group (coded as an indicator variable). Figure 3 indicates a movement of the density peak towards the left and upward with increasing currently alive age *a* that is more pronounced for the “high” activity group, which means there is a higher immediate death rate for this group. With increasing age *a*, the densities tend to have higher and sharper peaks, followed by a steadier decline. The global Fréchet regression models are fitted using the R function *GloDenReg* in the *frechet* package [29].

#### Analysis of continuous activity monitoring data

**Figure 4:**
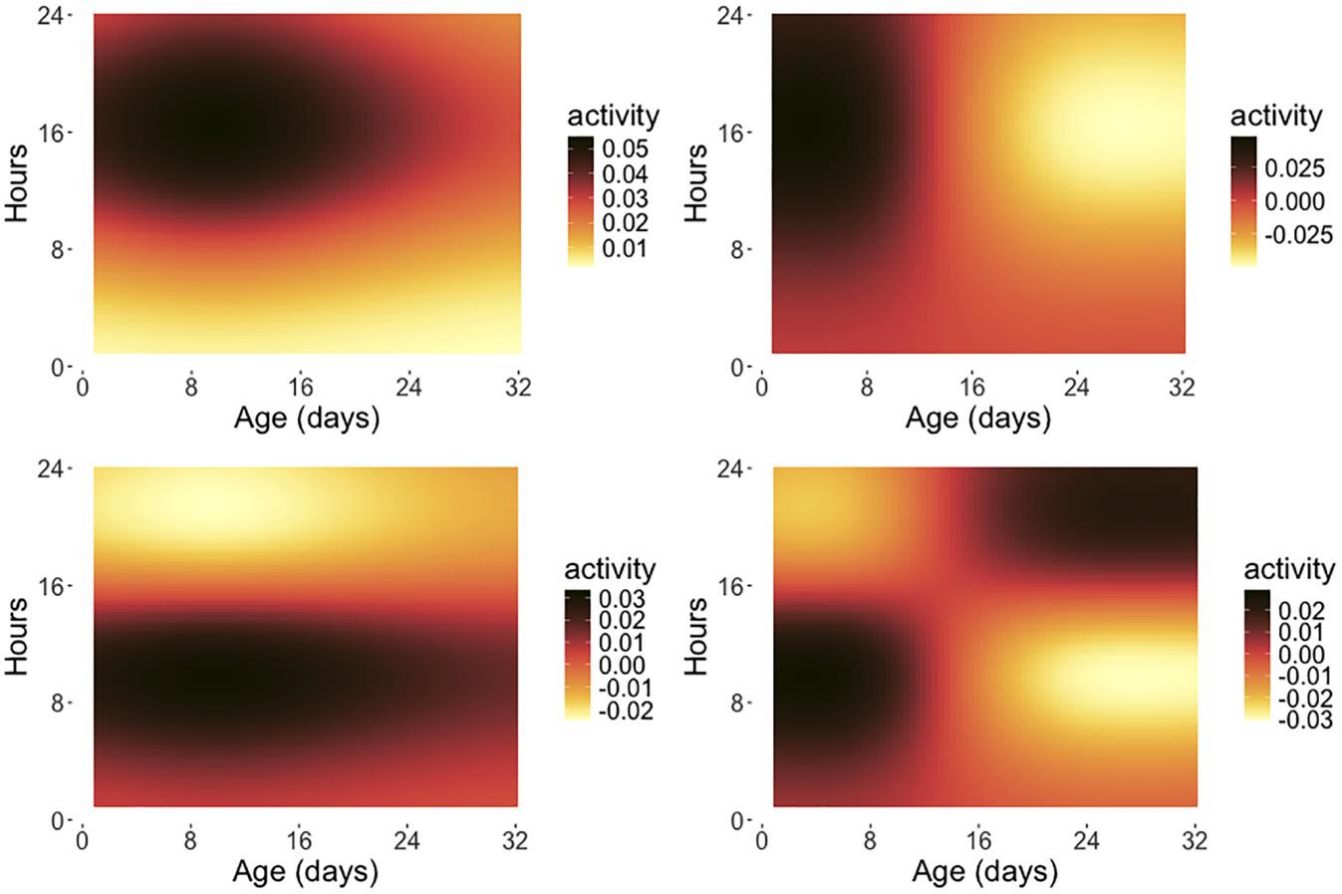
**Eigensurfaces for early age medfly activity data.** The first four eigensurfaces for product functional principal component analysis are depicted, with * ϕ*_1_(*t*)*ψ*_1_(*s*) (top left, 13.62%), * ϕ*_2_(*t*)*ψ*_1_(*s*) (top right, 8.62%), * ϕ*_1_(*t*)*ψ*_2_(*s*) (bottom left, 3.65%) and * ϕ*_2_(*t*)*ψ*_2_(*s*) (bottom right, 1.50%) as per (5), where the percentages represent the fraction of variance explained by the respective eigensurface and the * ϕ_j_* are the eigenfunctions reflecting the variation over the age span in days while the *ψ_k_* are the eigenfunctions for the intra-day variation. The x-axis indicates the age coordinate *t* (measured in days) and the y-axis represents the hour coordinate *s* within a given day (0–24 hours). Only flies surviving 32 days are included in this analysis.

Continuous activity monitoring data can be viewed as a function-valued stochastic process *X*(*s*, *t*), representing the activity count at age *t* (in days), which is the time index of the process and within-day hour *s*, which is the argument of the function observed at each age *t*. A key methodology is functional principal component analysis [30–32], which can be implemented with two-dimensional eigenfunctions [6] or, as we demonstrate here, with product eigenfunctions; for background see Chen et al. [14] and the Appendix). Figure 4 shows the first four eigenfunction surfaces for the product approach (see equation (5) in the Appendix), which is based on separate modeling of the hour and day dimensions, capturing potentially distinct patterns of variation for each of the day (age) and hour (within day) time scales.

The eigenfunction products delineate the main modes of variation for these processes, decomposing the total variation into interpretable components. Here the first eigenfunction surface * ϕ*_1_(*t*)*ψ*_1_(*s*) (upper left) illustrates a contrast between movement activity early (0–8 am) and late in the day (after 8 am), with diminishing contrast at older ages. The second eigenfunction surface* ϕ*_2_(*t*)*ψ*_1_(*s*) (upper right) highlights a contrasting pattern between activity at early and late ages (before and after 12 days), while the third eigenfunction surface * ϕ*_1_(*t*)*ψ*_2_(*s*) (bottom left) shows another contrast that divides the intra-day functions into periods before and after 4 pm. The fourth eigenfunction surface * ϕ*_2_(*t*)*ψ*_2_(*s*) (bottom right) emphasizes a reversal of the contrast between early and late activity at younger ages (before 4 pm until age 15 days) and late activity at older ages (after 4 pm beyond age 15), but overall does not explain much of the variation. Using the functional principal component scores for this product approach, one can further investigate the relationship between remaining lifetime and activity patterns; see [6] for more details. Implementations of these methods are available in the R package *fdarep* [33] via functions *Dense2dFPCA* and *DenseProductFPCA*.

### Reproduction and longevity

#### Cost of reproduction

The well-known cost of reproduction hypothesis in biodemography and life history analysis is still somewhat controversial due to a lack of clearly identifiable proximal factors. The general idea is that finite resources must be split between maintenance to extend lifespan and reproduction, both of which require protein sources [7, 34, 35], implying that high reproductive activity could be associated with reduced remaining lifespan. There is some evidence for a regulatory mechanism whereby lifespan is determined by remaining egg-laying potential; if this potential is low because eggs have been depleted then the remaining lifespan tends to be short and vice versa [17].

To study cost of reproduction related questions for cohorts of flies where reproduction can be measured as longitudinally measured daily egg-laying requires dedicated statistical methodology. We demonstrate this for a study of 473 female adult Mediterranean fruit flies who survived past age 35 days. The experiment was conducted at temperature 26 *±* 2°C, relative humidity 80 *±* 10%, and a 12:12 light:dark cycle. The medflies were placed in their own cages (6*.*5 *×* 6*.*5 *×* 12 cm plastic bottles kept horizontally) with a lid replaced by a fine mesh through which flies would lay their eggs that, in turn, would fall to a dish lined with a damp, black cloth. These oviposition dishes were collected daily for egg counting. On average, the daily number of eggs laid by female Mediterranean fruit flies tends to peak between 10 and 15 days after emergence and subsequently declines with age, as shown in previous analyses of these data [7, 17, 34].

**Figure 5:**
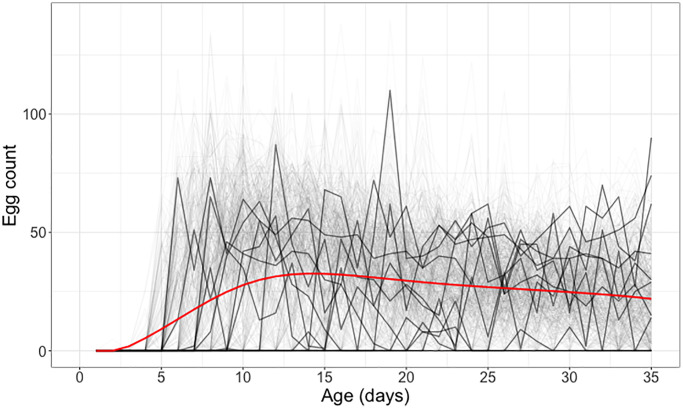
**Individual daily egg-laying counts for medflies surviving through 35 days.** Egg-laying counts for 10 randomly selected medflies are highlighted in black, while the other elements in the sample depicting individual daily egg-laying counts are shown in gray. The mean daily egg-laying count, obtained by local linear smoothing is shown in red.

The observed data are longitudinal daily egg counts, visualized in Figure 5. When analyzing such data, it is important to consider cohorts where all flies survive through a specified age *a*, in Figure 5 chosen as *a* = 35 days, to avoid censoring issues that will lead to bias. The figure shows that these data are afflicted by high variance, with high day-to-day variation.

#### Associations of daily egg count data and longevity

Elucidating the relationship between reproduction and longevity has been of long-standing interest in biodemography [7, 17, 36, 37]. Here we demonstrate the application of a global Cox point process regression model [15] for such data, where we use a reverse regression approach with age-at-death as predictor and the intensity function of the egg-laying process as response. While reversing the time order, this approach can serve to quantify and visualize associations between longevity and the egg-laying process and we implemented it with the function *GloPointPrReg* in the R package *frechet* [29]; technical details are in the Appendix. Additional tools include a graphical method to illustrate the connection between reproduction and longevity [38] and a forward prediction approach for remaining lifetime as response with the functional principal component scores obtained from longitudinally observed activity or reproductive trajectories up to current age alive *a* as predictors [6].

**Figure 6:**
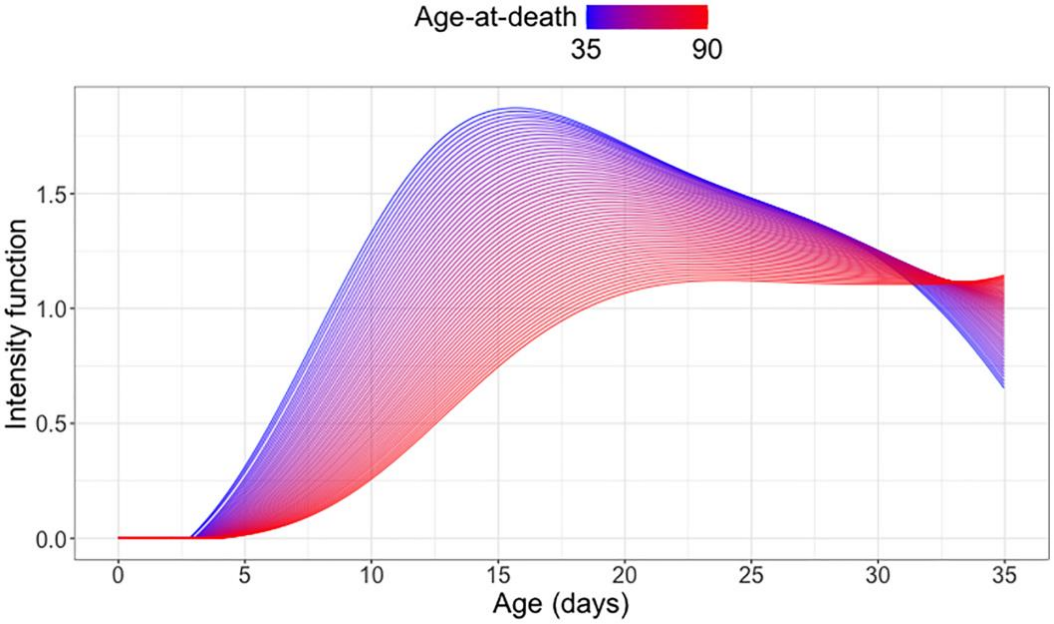
**Cost of reproduction: Quantifying the association between longevity and reproduction.** Predicted conditional intensity functions for daily egg-laying at different age-at-death levels ranging from 35 days (blue) to 90 days (red), using global Cox point process regression for a cohort of medflies who survived past 35 days (see (6) in the Appendix).

Figure 6 shows a distinct association between egg count trajectories and age at death. For medflies with lower age-at-death, the daily egg count tends to peak sharply around 15 days, with a steady decline thereafter. Flies who survive longer tend to have relatively lower egg counts at all ages until 30 days, and especially at younger ages, coupled with enhanced reproductive activity beyond 30 days after emergence. This pattern reflects the observed cost of reproduction for female medflies.

#### Predicting concurrent reproductive potential

How the current reproductive activity of a medfly at various ages relates to change in reproductive activity reveals the underlying dynamics of the reproductive process, complementing the dynamic model in 17]. Denote the egg-laying count on day *t* by *X*(*t*) with mean *µ_X_*(*t*) = *E*[*X*(*t*)] and consider difference quotients $Y(t)=\frac{X(t+1)–X(t)}{(t+1)–t}$, which serve to approximate the derivative (*d/dt*)*X*(*t*) that reflects reproductive activity change at age *t*. We aim to study the dependence of *Y*(*t*) on *X*(*t*) by fitting the concurrent regression model [18],


$$E[Y(t)|\;X(t)]=\beta_{0}(t)+\beta_{1}(t)(X(t)–\mu_{X}(t)),\;t\in [10,35]\;\;\;\;(1).$$

**Figure 7:**
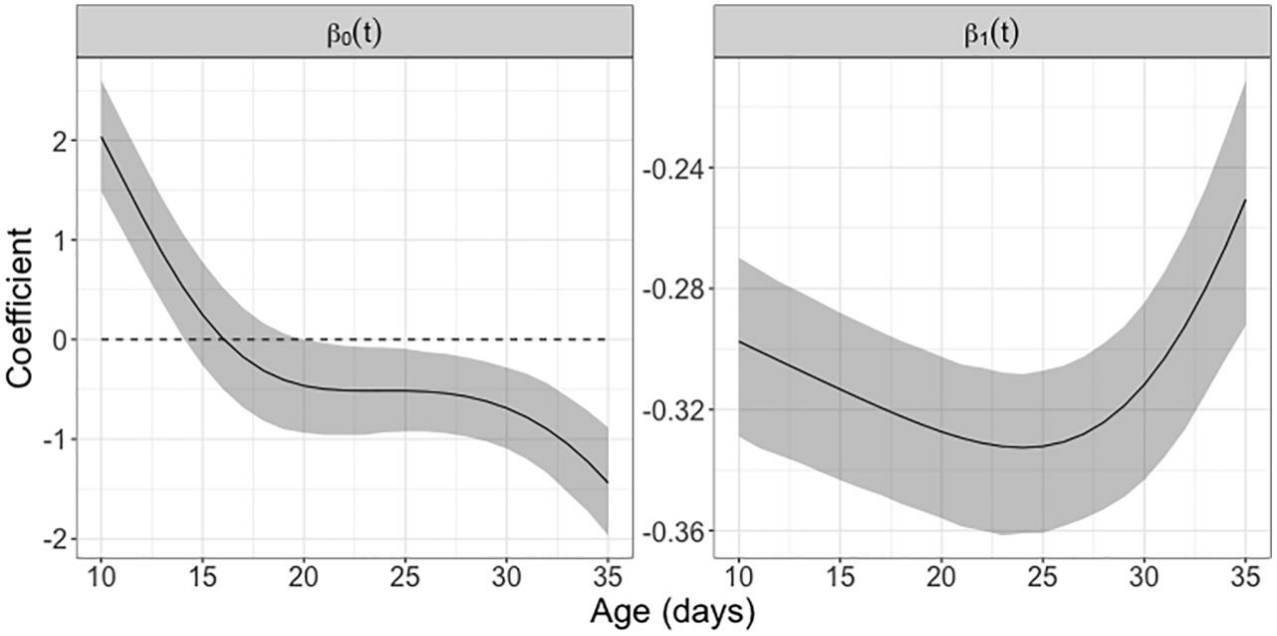
**Study of concurrent reproductive potential using varying coefficient model.** The intercept function *β*_0_(*t*) represents the (approximate) mean reproductive rate associated with daily egg-laying counts in female Mediterranean fruit flies that survived past 35 days. The regression coefficient function *β*_1_(*t*) depicts the time-varying effect of the accumulated egg-laying count on the immediate reproductive rate. The estimated coefficient functions are based on the fitted concurrent regression (1). The 95% pointwise confidence bands are based on 1000 bootstrap replicates, using concurrent regression (1). If zero is not located within the confidence band this indicates pointwise (not simultaneous) statistical significance in the corresponding age interval.

The estimated intercept function *β*_0_(*t*) represents the estimated mean derivative of the egg-laying trajectories. It is positive prior to 15 days, indicating that egg-laying is ramping up, then turns negative, indicating a decline in egg-laying that accelerates after 30 days, reflecting the depletion of the reproductive potential of female medflies (see Figure 7).

The estimated regression coefficient function *β*_1_(*t*) acts on the difference between an individual’s and the average egg-laying trajectory and takes negative values throughout; pointwise 95% bootstrap confidence intervals show that it is significantly negative from 10 to 35 days. This indicates a statistically significant effect whereby above-average egg-layers experience a more rapid decline than below-average egg-layers and conforms with a process that is self-regulating through a dynamic regression to the mean effect [39].

To fit the varying coefficient model and obtain the 95% bootstrap pointwise confidence bands, we used the functions *ConcurReg* and *GetCI Sparse*, available in the R package fdaconcur [40].

### Analysis of longitudinally monitored age-specific behavior patterns

#### Longitudinal compositional representation of medfly behaviors

For illustration, we use data on age-specific behavioral patterns continuously recorded for 51 Mediterranean fruit flies under controlled laboratory conditions (temperature: 25 *±* 2°C, relative humidity: 65 *±* 5%, light:dark cycle: 14:10). Each medfly was placed in an individual cage (12 *×* 5 *×* 7*.*5 cm transparent plastic cup). The behavioral patterns of each fruit fly were observed instantaneously 12 times each day through its lifetime (no censoring). In this analysis, we focus on a cohort of flies surviving past 41 days and the three behaviors of flying *Z*_1_(*t*), walking *Z*_2_(*t*) and resting *Z*_3_(*t*) at age, respectively; see [8] for further details.

In a preprocessing step, the three behaviors were transformed into proportions per time unit, where the proportions always sum up to 1 and are non-negative, thus forming compositional data; for example, 80% resting, 10% flying and 10%walking during one hour of observations, reflecting the observed proportions in time among just these three behaviors (other behaviors are not considered). We then obtained square-root transformed compositional proportions $X(t)=[\sqrt{Y_{1}(t)},\;\sqrt{Y_{2}(t)},\;\sqrt{Y_{3}(t)}],$ where $Y_{j}(t)=Z_{j}(t)/(Z_{1}(t)+Z_{2}(t)+Z_{3}(t)\;\text{for}\;j=1,\;2,\;3.$ For further details on compositional data and their representation on the positive orthant of a sphere, see [41–43]. This approach then leads to longitudinal data that are situated on a sphere.

#### Spherical functional principal component analysis for longitudinal behavior data

Following [22] and [44], we first map the data to linear tangent spaces using Riemannian log maps centered at the Fréchet mean curve and then carry out a regular multivariate functional principal component analysis on the linear tangent space of the log-mapped data; spherical functional principal components, eigenfunctions and finite-truncated representations of the log-mapped data are first obtained on the tangent space and then mapped back to the original spherical space by applying Riemannian exp maps. An implementation is available through the R function *RFPCA*, available on GitHub at https://github.com/CrossD/RFPCA.

**Figure 8:**
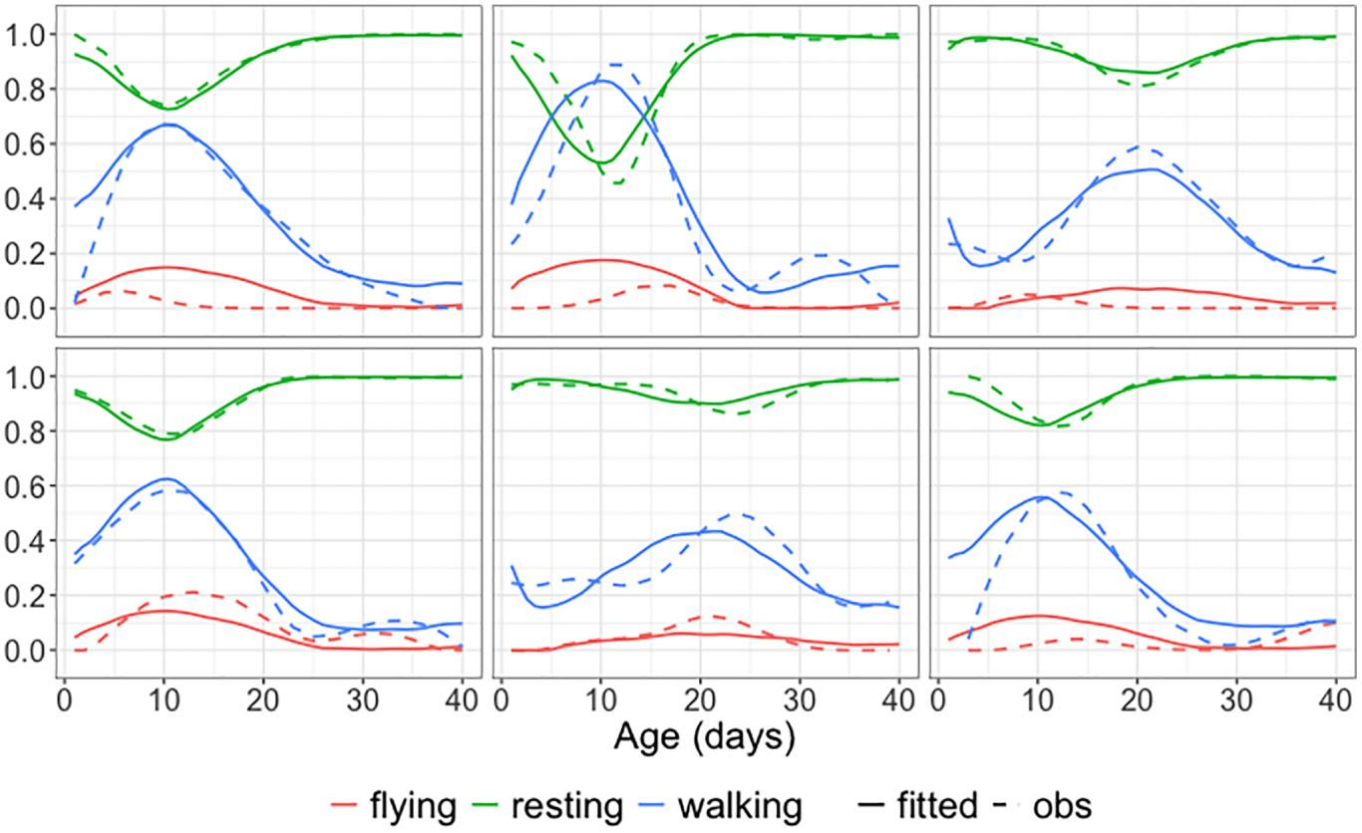
**Observed and fitted trajectories for age-varying behavioral data.** Observed data (solid lines) and spherical functional principal component analysis fitted behavioral trajectories (dashed lines) for six randomly selected medflies, with three selected components. The close alignment demonstrates a good fit.

**Figure 9:**
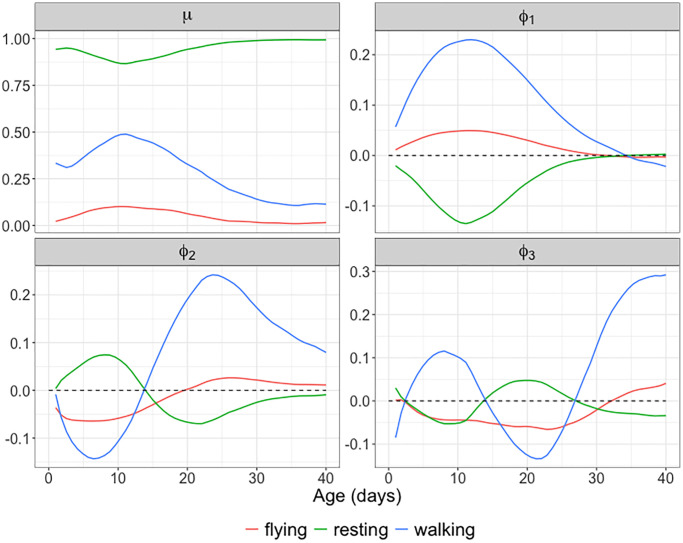
**Mean function and eigenfunctions for longitudinal behavioral data.** The estimated mean function $\hat{\mu }$ and the first three estimated spherical eigenfunctions ${\hat{\phi }}_{1},\;{\hat{\phi }}_{2},\;{\hat{\phi }}_{3}$ for the behavioral fly data using spherical functional principal component analysis. The first three eigenfunctions explain 96.54% of the total variation, with the individual components explaining 80.55%, 12.23% and 3.76%, respectively.

Figure 8 displays the observed and fitted trajectories *X*(*t*) for six randomly selected medflies, using the first three components obtained for the spherical functional principal component analysis. The close alignment between fitted and observed trajectories indicates a good fit. Figure 9 further illustrates the mean function and the first three eigenfunctions; each of these consists of three functions corresponding to the three behaviors. Figure 9 indicates that resting and walking were commonly observed, while flying occurred more rarely.

The first three eigen components explain more than 96% of the total variation. The first eigenfunction reflects an overall contrast between resting and moving (flying or walking) throughout the observation period. The second eigenfunction reflects a contrast between early (0–15 days) and late (16–40 days) age periods, while the third eigenfunction features a contrast for both resting and walking between the middle age period (15–25 days) and the early/late age periods. Invoking modes of variation through this approach facilitates a comprehensive analysis of complex, time-varying behavioral patterns.

### Software

Several R packages are available for computational implementations of the methods illustrated in this perspective and beyond.

The R package *fdapace* [45] provides implementations of Functional Data Analysis and Empirical Dynamics. Central to this package is Functional Principal Component Analysis, a key dimension reduction tool for functional data analysis, used for both sparsely and densely sampled random trajectories and time courses via the Principal Analysis by Conditional Estimation (PACE) algorithm. This core algorithm yields covariance and mean functions, eigenfunctions and principal component scores for functional data and their derivatives, covering both dense (functional) as well as sparse (longitudinal) sampling designs. Additionally, it provides fitted continuous trajectories with confidence bands for sparse designs, even for subjects with very few longitudinal observations, thereby presenting a viable and flexible alternative to more classical random effects modeling of longitudinal data. There is also a Matlab version (PACE) [25, 26, 46, 47].

The R package *frechet* [29] provides an implementation of statistical methods for random objects in various metric spaces. The core functionality of this package is Fréchet regression for random objects with Euclidean predictors, enabling regression analysis for non-Euclidean responses under mild conditions. As a special case, *frechet* includes the implementation of global and local Cox point process regression with point processes as responses and an intrinsically non-parametric intensity function [15]. This package is particularly useful for handling data such as distributions in 2-Wasserstein space, covariance matrices endowed with power metrics (including the Frobenius metric as a special case), and spherical data, among other data types [10].

The R package *fdaconcur* [40] provides an implementation of concurrent or varying coefficient regression methods for functional data, accommodating both densely and sparsely observed data. It includes the construction of pointwise confidence bands and models the influence of past predictor values through a smooth history index function [18].

The R package *fdarep* [33] is designed for repeatedly observed and thus dependent functional data, aiming at a framework where curves are recorded repeatedly for each subject in a sample. In particular, fdarep provides an implementation of two-dimensional functional principal component analysis (FPCA), Marginal FPCA, and Product FPCA for repeated functional data [14, 48].

## DISCUSSION

While we focus here on longitudinal data that relate to movement activity, egg-laying activity and behaviors that are continuously monitored, other longitudinal trajectories and their relationship with remaining lifetime distributions could be of equal interest. One issue is that one may encounter missing data or irregular designs with sporadic rather than continuous monitoring. Such irregular designs can be handled with the same methods as described above, and the software we refer to is also sufficiently flexible to handle irregularly recorded data. This is exemplified in [49], where functional data analysis methodology is shown to be superior to classical random effects models that traditionally have been used to handle missing or irregular and sparsely sampled longitudinal data. Various alternative implementations of functional principal component analysis are also available [50, 51].

Another monitoring design that is sometimes encountered generates functional snippets, where individuals are continuously monitored only over a subject-specific limited range of ages that is a subset of their lifespan, but not over the entire lifespan. This scenario is common for human data, where often only a relatively short period of monitoring is feasible and in any study where budgetary or logistic limitations allow only for a time-limited monitoring period. The age when an individual enters the monitoring period varies by subject, as subjects of all ages are enrolled at inception of the study. Since the resulting functional snippet data convey less information compared to longitudinal monitoring over the entire lifespan and do not allow for direct auto-covariance estimation, which is a fundamental step for functional data analysis [13], only more restrictive approaches are available that require additional prior information and stronger assumptions [52–54].

For human data, the level and impact of movement activity on various health conditions was the topic of various studies [55–57]. Special care is needed to avoid cofounding with factors such as socio-economic background that may bias the conclusions. Human studies have been predominantly cross-sectional rather than longitudinal and thus their emphasis is on cohort mean effects rather than individual random effects, where the latter is the emphasis of this article; for the relation between individual and cohort longevity see [58].

The statistical methodology illustrated in this article, centered around longitudinal and functional data analysis, distributional data analysis [59] and the more general framework of metric statistics and Fréchet regression [60], has also been employed for the analysis of samples of mortality trajectories (without monitoring data) [61–63]. Such data can be viewed as realizations of a stochastic mortality process and are easily obtained from various sources, including the Human Mortality Database (Max Planck Institute for Demographic Research, available at http://www.mortality.org).

### Data availability statement

The data supporting this study’s findings are available from James R. Carey upon reasonable request (email address: jrcarey@ucdavis.edu).

## Appendix

### Global Fréchet regression

We apply global Fréchet regression [10] with the remaining lifetime distribution observed at a given current alive age *a* as response. Here the space of distributions *W* is equipped with the 2-Wasserstein distance


$$d_{W}^{2}(F_{1},F_{2})=\int_{0}^{1}{\left\{F_{1}^{-1}(u)-F_{2}^{-1}(u)\right\}}^{2}\;du,\;\;F_{1},\;F_{2}\in W$$

where $F_{1}^{-1}$ and $F_{2}^{-1}$ are the corresponding quantile functions of distribution functions *F*_1_ and *F*_2_. Global Fréchet regression aims at conditional Fréchet means,


$$F_{x,⊕}=\underset{\upsilon \in w}{\text{argmin}}\;E[d_{W}^{2}(F,\;v)|X=x].\;\;\;\;(2)$$

Assume the random pairs ${\left\{(X_{i},F_{i})\right\}}_{i=1}^{n}$ are realizations of (*X*, *F*), where *X* is a scalar or vector predictor. In practice, we estimate the remaining lifetime distribution *F_i_* from the sample of surviving medflies. The global Fréchet regression estimate is


$${\hat{F}}_{x,⊕}=\underset{\upsilon \in w}{\text{argmin}}\;\frac{1}{n}\sum_{i=1}^{n}\hat{s}(X_{i},\;x)d_{W}^{2}({\hat{F}}_{i},\;v),\;\;\;\;(3)$$

where $\hat{s}(X_{i},x)=1+{\left(X_{i}-\bar{X}\right)}^{T}{\hat{\Sigma }}^{-1}(x-\bar{X}),\;\bar{X}=n^{-1}\Sigma_{i=1}^{n}\;X_{i}$ $\text{and}\;\hat{\Sigma }=n^{-1}\;\Sigma_{i=1}^{n}(X_{i}-\bar{X}){\left(X_{i}-\bar{X}\right)}^{T}$ are weights derived from a linear regression model. One can then obtain estimates for density and hazard function of the remaining lifetime distribution,


$${\hat{f}}_{x,⊕}(u)=\frac{\partial {\hat{F}}_{x,⊕}(u)}{\partial u},\;{\hat{\lambda }}_{x,⊕}(u)=\frac{{\hat{f}}_{x,⊕}(u)}{1-{\hat{F}}_{x,⊕}(u)},\;u\in (0,\;90).\;\;\;\;(4)$$

### Product functional principal component analysis

Product functional principal component analysis (FPCA) serves as a dimension reduction tool for a function-valued stochastic process. If *X*(*s*, *t*) denote the activity level at hour *s* ∈ *S* = [0, 24] within day *t* ∈* T* = [0, 32], the mean and covariance functions of the underlying process are μ(s, t)=E[X(s, t)] and $G((s_{1},t_{1}),(s_{2},t_{2}))=E[X(s_{1},t_{1})X(s_{2},t_{2})-\mu (s_{1},t_{1})\mu (s_{2},t_{2})],$ $s_{1},s_{2}\in S,\;t_{1},t_{2}\in T.$

Writing ${\text{\{}\psi_{j}(s)\text{\}}}_{j=1}^{\infty }$ for the eigenfunctions of the operator in *L*^2^(*S*) with marginal kernel *G_S_*(*s*,*u*) = $\int_{0}^{32}G((s,t),(u,t))dt$ and ${\text{\{}\phi_{k}(t)\text{\}}}_{k=1}^{\infty }$ for the eigenfunctions of the operator in *L*^2^(*T*) with marginal kernel $G_{T}(t,u)=\int_{0}^{24}G((s,t),(s,u))ds,$ one can represent *X*(*s*, *t*) through a product FPCA as follows,


$$X(s,t)-\mu (s,t)=\sum_{j=1}^{\infty }\sum_{k=1}^{\infty }\chi_{jk}\phi_{k}(t)\psi_{j}(s).\;\;\;\;(5)$$

Here

$$\chi_{\mathrm{jk}}=\int_{0}^{32}\int_{0}^{24}\left(X(s,t\right)-\mu (s,t))\psi_{j}(s)\phi_{k}(t)dsdt$$

are the principal component scores that are used to summarize the two-dimensional process. These components are zero mean uncorrelated random variables representing the fluctuations of the process *X*(*s*, *t*) around the mean function *µ*(*s*, *t*).

### Global Cox point process model

Writing *N*(*t*) for the point process that represents egg-laying at age *t*, we model (*N,* Λ) as a doubly stochastic Poisson process, where we postulate an underlying stochastic (positive integrable) intensity process Λ(*t*), such that *N*|Λ = *λ* is a non-homogeneous Poisson process with intensity function *λ* [15, 64]. Given a realization of the latent process Λ = *λ*, the expected number of eggs laid up to time *t* is *E*[*N*(*t*)|Λ = *λ*] = $\int_{0}^{t}\lambda (u)du.$ Conditional on observing *N*(*T*) = *m* > 0 events with associated intensity function Λ = *λ*, the random event times *T*_1_, … *T_m_* at which egg-laying occurs are independently and identically distributed.

To regress the infinite-dimensional object Λ on age-at-death *X*, we fit a global Cox point process regression model, targeting


$$m_{⊕}(x)={\text{argmin}}_{\lambda \in \Omega }\;E[d^{2}(\Lambda ,\lambda )|X=x],\;\;\;\;(6)$$

where (Ω, *d*) forms a metric space of intensity functions and Λ ∈ Ω represents the random intensity function of egg-laying events. Intensity functions Λ ∈ Ω admit a one-to-one decomposition into two components, namely, the intensity factor $\tau =\int_{0}^{T}\Lambda (s)ds$ and the density function $f(t)=\frac{\Lambda }{\tau }.$ Thus, the intensity function space Ω can be viewed as the product metric space Ω =D × Ω_*s*_, where *D* denotes the space of density functions over [0, *T*] and Ω_*s*_ = (0, ∞) denotes the space of intensity factors. We utilize the *l*^2^ type product metric *d* between intensities $\Lambda_{1}=(f_{1},\tau_{1})\;\text{and}\;\Lambda_{2}=(f_{2},\tau_{2}),$ which is given by


$$d(\Lambda_{1},\Lambda_{2})={\left(d_{W}^{2}(f_{1},f_{2})+d_{E}^{2}(\tau_{1},\tau_{2})\right)}^{1/2},$$

Here *d_E_* is the Euclidean metric and *d_W_* is the Wasserstein metric between probability distributions. While the intensity function of egg-laying events remains unobservable, its components, namely the density function and the intensity factor, can be estimated from available data.
